# The Term Equity in Education: A Literature Review with Scientific Mapping in Web of Science

**DOI:** 10.3390/ijerph17103526

**Published:** 2020-05-18

**Authors:** Pedro Jurado de los Santos, Antonio-José Moreno-Guerrero, José-Antonio Marín-Marín, Rebeca Soler Costa

**Affiliations:** 1 Department of Applied Pedagogy, Autonomous University of Barcelona, 08193 Barcelona, Spain; pedro.jurado@uab.cat; 2 Department of Didactics and School Organization, University of Granada, 18001 Granada, Spain; jmarin@ugr.es; 3 Department of Educational Sciences, University of Zaragoza, 50009 Zaragoza, Spain; rsoler@unizar.es

**Keywords:** scientific production, bibliometric analysis, scientific mapping, equity, education, Web of Science

## Abstract

The term “equity” (EQUI) is a complex concept to be defined, because it depends on many factors, mainly political ideals. The objective of this research is to determine the evolution and development of the term equity in education by the scientific community. The main objective is to identify the scientific production and performance of the term equity in the field of education. The research method developed is based on bibliometrics, specifically on the technique of scientific mapping, and a process of quantification, analysis, evaluation and estimation of scientific documents was developed. The results indicate there is no established line of research or strong connections between the themes. This shows the existing variety of research on the term equity. Thus, the trend in research on the term equity is focused on the early periods of study on the students’ diversity in order to evolve to more concrete and specific aspects of equity, such as gender and race. It is concluded that the theoretical framework of reference shows how equity should be incorporated into the education system under the parameters of equal opportunities, of equality in access to higher education, regardless of gender or socio-cultural background.

## 1. Introduction

The first reference made to the term “equity” was related to the need established by many countries, in 1990, to bring together two terms—equity and quality—in the supply of education in each nation. Many more countries have since joined this endeavor, beginning in 2004. The conceptualization of the term is twofold: on the one hand, those normative references that confirm the fact that the principles of quality and equity are inseparable. This conceptualization of equity is based on two principles: quality education for all citizens, seeking to develop to the maximum the individual, social, intellectual, cultural, and emotional capacities, always within a framework of effective equality of opportunity; and the shared effort of the entire education community in caring for the diversity of students. On the other hand, the term equity is associated with social well-being, based on the principle of personalized and universal education. In this case, equity and quality are considered to be two sides of the same coin, as well as making reference to the fact that if there is laziness and mediocrity, there will be no equity. The researchers themselves present lines of research focused on aspects directly related to equity, such as attention to diversity. This study seeks to analyze how the term equity is treated, in the area of education, by the scientific community. In doing so, it seeks to offer a more concrete and specific perspective, both to the educational community and to the scientific community, for understanding the approaches to and scope of the term equity.

### Justification and Objectives

It is important to note that the term equity is different from equality. More precisely, along this heading we will present the basic principles and characteristics of each one of the educational conceptions that can be assumed [1,2]. On the one hand, equality refers to having the same resources and opportunities, that is to say, to satisfy the needs of each individual. This principle is a long-term objective of a just society where children, regardless of ethnic origin, socio-economic class, or gender, should have access to the same resources and opportunities. In addition to these egalitarian aspects, equality must be made effective in the treatment and non-discrimination of people with disabilities, also promoting non-sexist and non-stereotypical attitudes. It will be clear that we have achieved this goal when schooling and economic success and opportunity are equal for all groups of children.

On the other hand, by equity we mean a system where common goods are redistributed to create systems and schools that share a greater likelihood of being more equal [1]. This educational approach manifests itself in an equitable system where additional resources are provided so that students have the opportunity to excel academically and socially. From this vision, equity requires an unequal distribution of resources in the hope that sustained equity will temporarily favor and promote more equal educational opportunities for students, in which all reach the maximum possible development of their individual and social, intellectual, cultural and emotional capacities. Students should receive a quality education adapted to their needs, thus making equity and quality the two sides of the same coin [3]. This premise would imply that differences between people would not be a risk factor for discrimination, exclusion, or social, labor or educational disadvantage, but rather an opportunity to improve them [4] and to meet their needs. These approaches can promote egalitarian thinking on student performance and achievement when inequality in this sense is not necessarily unfair, as differences in student outcomes may be due to differences in students’ efforts, motivations, interests, talents, or even luck [2].

Currently, education systems have equity as one of their guiding principles [4,5,6]. This fact manifests itself in different ways according to the ideologies underlying the educational policies that are implemented [7]. From a neoliberal point of view, equity can be understood as the right to receive training according to one’s possibilities, which allows one to choose a professional path and enter the world of work [8]. In this way, education is utilitarian and becomes a right that has as its main objective the formation of future citizens as a workforce [6]. Thus, the education system is an accomplice to the perpetuation of social inequalities without reducing the initial disadvantages. On the other hand, equity is conceived in the education system as part of social justice in which each person, by virtue of being a person, receives what he or she needs from the common goods to compensate for the initial inequalities and, thus, annul the biases related to personal, social, or cultural factors [9].

Equity in education should not only be seen as compensation or readjustment of common goods in order to alleviate initial limitations, but should also go beyond this by seeking to ensure that this equity is manifested in such important aspects as equal opportunities in access to studies regardless of gender [10,11], social origin [12,13], or ethnic origin [12,13,14]. It also seeks to ensure that this equity is reflected in academic results and quality [15], which will allow students to access higher education and thus break the inequality gap [16]. 

There are various measures to promote equity in education. Examples are educational resources, financial aid for study, and measures to address diversity. [4]. There are also other measures to promote equity. One such measure is the cultural and organizational organization of educational centers [17,18,19,20,21]. Schools must promote routines and practices that encourage educational leadership. This leadership should favor the entire educational community, focusing mainly on teachers, parents, and students [17]. In this sense, educational leadership [6,13] appears as a dynamic element of educational practices based on equity and social justice. In addition, collective work [5] emerges as a central principle to work on. It favors transforming and liberating change in teaching, learning, schooling, education, and society.

For this equity to be effective in schools, teachers must be involved and made aware of the need to implement strategies and actions [22] that promote equity [23] and, in turn, mobilize the different agents in the community where the school is located [20] to actively participate in the fight against inequalities [24]. In this sense, recent research [25] has revealed how schools working in the most disadvantaged contexts become the most powerful means of promoting improvements through school partnerships.

The importance of working for a more equitable education system is reflected in the reports on this subject that are being produced in different countries [2] and the proposal that the United Nations launched through the Agenda 2030 on Sustainable Development [26] with its 17 Sustainable Development Goals. Objective 4, dedicated to quality education, expressly mentions the need to guarantee inclusive, equitable, and quality education that produces relevant and effective learning outcomes. It is clear from these initiatives that, in order for these changes to be effective in society and, therefore, in the education system, there must be effective and sustainable involvement of all social actors, that is, the public and private sectors, microenterprises, and cooperatives, as well as multinationals, civil society organizations, and philanthropic organizations [26]. Only through the personal involvement of each citizen and the collaboration of other organizational structures can we work to provide safe, non-violent, inclusive, and effective learning environments for all, and thus achieve effective equity in education.

To develop this research, the term “equity” was analyzed in articles in the Web of Science (WoS) database. This database is owned by Clarivate Analytics. It contains a collection of databases of bibliographic references and citations to periodicals that collect information from 1900 to the present. The WOS is composed of the Core Collection which covers the Science, Social Science, and Arts and Humanities indexes, as well as the Proceedings of both Science and Social Science and Humanities along with tools for analysis and evaluation, such as the Journal Citation Report and Essential Science Indicators. Additionally, it comprises databases that complement it and are included in the license for Spain: Medline, Scielo, and Korean Citation Index. Because of the high relevance of this database, the authors selected it to conduct the present study. The research has a scientific mapping, considering some bibliometric indicators and the analysis of the structure and dynamism of terms. For this purpose, the analytical structure of previous studies taken from the Journal Citation Reports (JCR) [27,28,29] has been assumed. This provides a model already accepted by the scientific community. 

This study, for the Special Issue “Transculturality, Education and Health in the Digital Age and Times of Uncertainty”, aims to analyze the term equity in the scientific field. This term is of utmost importance. If it is not well established at the educational level, it can generate situations of social exclusion, and therefore, generate public health problems. This is a reality, given that public health is not an isolated element or disconnected from social realities.

The aim of this research is to show the evolution of the term equity in the publications of the WoS main collection. There are several reasons for this, one being the gap between the scientific literature on the state of the art. Moreover, there is no study analyzed with this bibliometric technique of documentary analysis. This study profile aims to identify the main lines of research on the term equity. In this sense, this research explores a specific area of knowledge to offer the scientific community new discoveries about the delimited construct. This implies, among other things, a great evolution of science and the satisfaction of the knowledge needs of researchers in this field. Therefore, with this study we try to identify the specific and concrete profile of the term equity in the educational field.

To do so, it is necessary to keep in mind a series of specific objectives: (a) to know the performance of scientific production on equity in the field of education; (b) to analyze the scientific evolution of equity in the field of education; (c) to detect the most incidental questions on equity in the field of education; and (d) to identify the most influential authors on equity in the scientific literature of the field of education.

## 2. Materials and Method

### 2.1. Research Design

The research methodology to achieve the formulated objectives was bibliometrics, which can be understood as the branch of scientometrics that analyzes scientific publications. The methodological branch assumed is based on the potentialities of scientometrics, which can be defined as the statistical and sociometric analysis of the scientific literature through the use of mathematical models, in questions related to the processes of searching, recording, analyzing, and predicting the academic literature [30]. This research was developed on the basis of the guidance of experts in this method of study [31].

More specifically, this research was based on an analysis of co-words [32] and of various bibliometric indicators and indexes (h, g, hg, q2) [33]. These data allowed attainment of a set of maps with nodes that will show the performance and the location of sub-domains of the constructs connected to “equity” (EQUI). In addition, the graphic preparation will facilitate the development of the themes on EQUI in the database initially established [34].

### 2.2. Procedure

**Figure 1:**
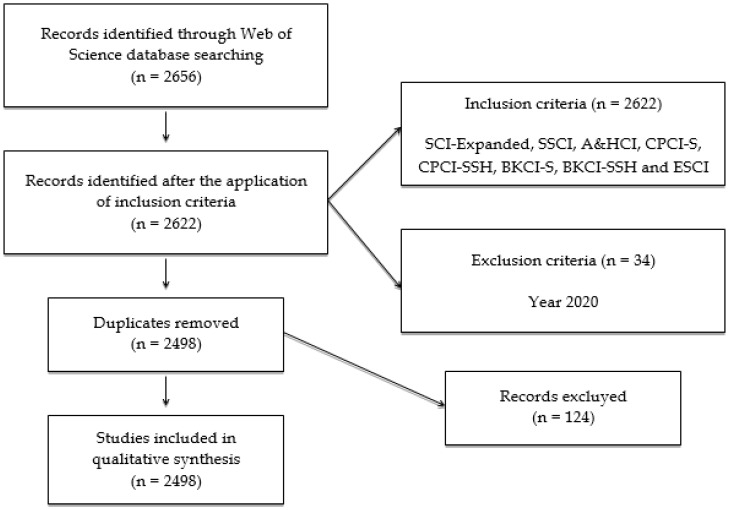
Flowchart according to the PRISMA declaration.

This investigation followed several processes: (1) choice of the database to be analyzed (WoS); (2) determination of the key words to be considered (“equity”); (3) elaboration of the search equation (“equity” TITLE in the categories of “Education Educational Research”, “Education Scientific Disciplines”, “Psychology Educational”, and “Education Special”); and (4) selection of the search process by bringing together the TOPIC process to report documents that included the concept to be analyzed in metadata comprising the title, abstract, and key words. This action allowed access to a first data report of 2656 publications. Thus, the concept of equity was first found in the scientific literature in 1948. In this sense, this research covered the analysis of a literary volume of 71 years (until 2019 inclusive). Publications relating to 2020 (n = 34) were eliminated because the calendar year was not completed. In addition, repeated or improperly indexed documents were deleted (n = 124). This resulted in a final unit of analysis of 2498 documents. These actions are reflected in the following flow chart, taking into consideration the protocols of the PRISMA-P matrix (Figure 1).

Thus, this volume of production was derived from the application of certain production indicators and inclusion criteria (year of publication = all production except 2020; language ≥ 10; publication area ≥ 45; type of documents ≥ 200; organizations ≥ 45; authors ≥ 8; sources of origin ≥ 30; countries ≥ 100; citation (the four most cited documents) ≥ 152.

### 2.3. Data Analysis

**Figure 2:**
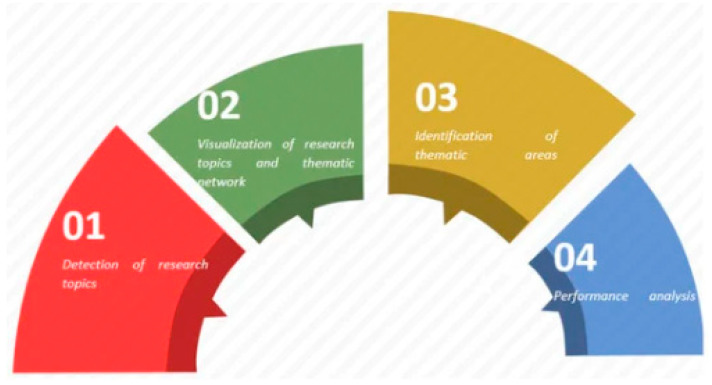
Phases of co-word analysis using SciMAT. Recovered from [36].

In order to determine the year, authors, country, type of document, institution, language, media, and most cited documents of the resulting production, “Analyze Results” and “Creation Citation Reports” (tools integrated in the WoS platform) were used. The SciMAT (Science Mapping Analysis Software Tool), is a free and open source tool. It is used to perform scientific map analysis. It integrates the strengths of other bibliometric tools (CoPalRed, BibExcel, VOSViewer, etc.), as well as reduces dependence on external tools. This software integrates everything needed to perform scientific map analysis, in a longitudinal framework and through bibliometric impact measurements. It also allows the analysis of the social, intellectual, and conceptual evolution of a research field. SciMAT was used to analyze the structural and dynamic development, following the recommendations of the specialists [35], for an effective co-word analysis, in which the following phases were carried out (Figure 2):

- Recognition: in this phase, the keywords of the documents are examined (n = 3513), a map of co-occurrence nodes is created, a standardized network of co-words is built, the most significant keywords are detected (n = 3331), and the most incidental topics and concepts are represented by means of a clustering algorithm.
- Reproduction: at this point, a strategic diagram and a thematic network are built based on the principles of centrality and density. The graphs derived from this have four zones: upper right = motor and relevant themes; upper left = rooted and isolated themes; lower left = disappearing or growing themes; lower right = underdeveloped and cross-cutting themes.
- Determination: in this phase the evolution of the nodes distributed in several periods or intervals of time is analyzed. Five periods were articulated in this study (P_1_ = 1948–2006; P_2_ = 2007–2011; P_3_ = 2012–2016; P_4_ = 2017–2019). However, for authorship, only one period was configured which covered the full period studied (PX = 1948–2019). To find the strength of association, the number of common keywords in the different periods is taken into account.
- Performance: in this phase, several production indicators configured with different inclusion criteria were applied (Table 1).

## 3. Results

### 3.1. Scientific Performance and Production

**Figure 3:**
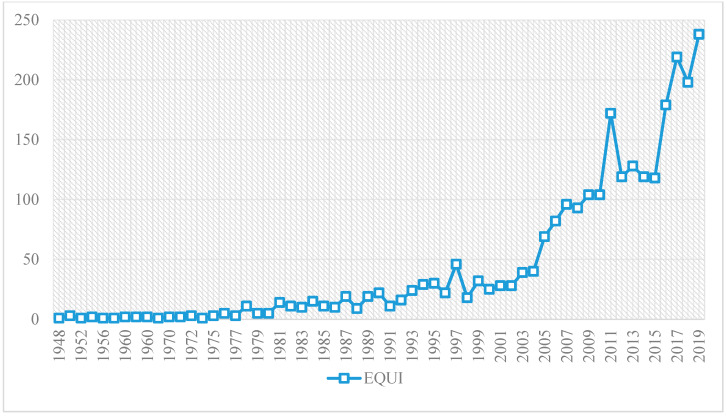
Evolution of scientific production.

The 2498 documents analyzed on the production of the term equity represented all scientific output from 1948 to 2019, both inclusive. As shown in Figure 3, the evolution is irregular since its beginning. From 1948 to 2004, scientific production is scarce, never exceeding 50 documents per year. From 2005 onwards, there is an exponential growth until 2011, although in 2012 the production decreases abruptly, being constant in terms of the number of publications until 2015, when it grows until 2017, interrupted by a small recess in 2018, before growing again in 2019. 

The language used by the scientific community to present the results of their research is English, followed, by a significant margin, by Spanish (Table 2).

The main knowledge area where equity studies are collected is Education Educational Research, followed, by a significant margin, by Education Scientific Disciplines (Table 3).

The article is the main type of document used by the scientific community to present their results. Table 4 also highlights the high level of book chapter production, which is unusual in many bibliometric studies.

Among all the existing institutions that carry out studies related to equity, the University of California System stands out, followed at a considerable distance by the California State University System (Table 5). 

The most productive author on equity studies is Kyriakides, L., followed closely by Charalambous, E. (Table 6).

There are two sources that are considered to be the reference sources for publication of EQUI. In this case they are Teacher College Record and Phi Delpa Kappa (Table 7).

The most relevant country in the production of EQUI is the United States. The next country is England, although it significantly lags the US (Table 8). 

The most cited publication in equity studies is that developed by Warschauer and Matuchniak (2010), with a total of 231 citations. It is followed by the study by Nieto (2000), with a total of 199 citations (Table 9).

### 3.2. Structural and Thematic Development

**Figure 4:**
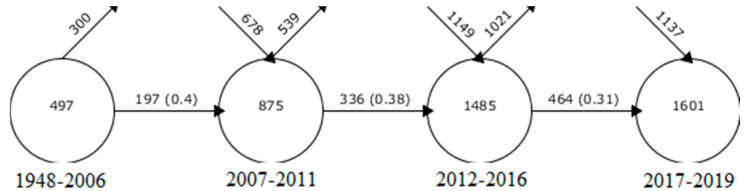
Continuity of keywords between contiguous intervals.

The keyword continuity between contiguous intervals shows the number of keywords entering and leaving a given period, and also provides information on the percentage of keyword matching between established periods. As shown in Figure 4, the level of keyword matching between periods is decreasing from the beginning to the present. This shows new trends in the field of equity studies in recent times. Although the distribution of documents by the established periods has been even, an irregular level of keyword matching is shown. This is due to the years collected, since the use of keywords was not widespread in scientific production. 

The academic performance of the established periods presents the most relevant themes and those with the highest values in the bibliometric indices, among which are the h index, the g index, the hg index, and the q^2^ index. In the first period (1948–2006), the subject with the highest bibliometric value is “diversity”, followed by “students”. In the second period (2007–2011), “equity” has the highest value, followed by “mathematics”. In the third period (2012–2016), there are two themes with equal bibliometric levels, namely, “policy” and “students”. In the last period (2017–2019), the bibliometric levels are evenly distributed, highlighting in this case the themes “race”, “achievement”, and “gender” (Table 10).

The interval diagram tries to show the level of importance of each of the analyzed topics in the thematic performance. To do this, it takes into account a process of grouping, using the Callon index. This indicator analyses the degree of interaction of a network with respect to other networks, from two perspectives: centrality, which measures the strength of external links with other topics, being the measure of the importance of a topic in the development of a certain field of research; and density, which analyzes the internal strength of the network, identifying the internal links between all the key words that are grouped around a specific topic, thus offering the degree of development of the field of study analyzed. The analysis of the four established diagrams does not show a theme that is the driving force in the four periods. 

In the first period (1948–2006), the driving themes are “sex-differences”, which deals with “girls”, “attitudes”, “sex-equity”, “classroom”, “gender-differences”, “teacher-student-interactions”, “race”, and “achievement”; and “diversity”, which refers to “educational-equity”, “multicultural-education”, “preparing-teachers”, “perspectives”, “equity”, “social-justice”, “reform”, and “teacher-education”. During this period, research focuses on aspects directly related to gender differences and equity in education.

In the second period (2007–2011), the driving themes are “mathematics”, which deals with “inquiry”, “principal-leadership”, “student-achievement”, “elementary-schools”, “high-school”, “self-efficacy”, “pedagogy”, and “achievement”; and “gender”, which refers to “class”, “ethnicity”, “internet”, “teachers”, “women”, “race”, and “science-education”. In this period, research focuses mainly on mathematics, and the gender of people.

In the third period (2012–2016), the driving themes are “gender”, which deals with “culture”, “boys”, “values”, “adolescents”, “impact”, “women”, “politics”, and “girls”; “social-justice”, which refers to “national-norms-and-standards”, “school-fees”, “no-fee-schools”, “standards”, “principal-role”, “urban”, and “funding”; and “race”, which relates to “white”, “choice”, “stratification”, “admissions”, “university”, “engagement”, “access”, and “faculty”. In this period, gender is again taken as a reference for research. In addition, new terms appear as relevant, as is the case of social justice and the races of people.

**Figure 5:**
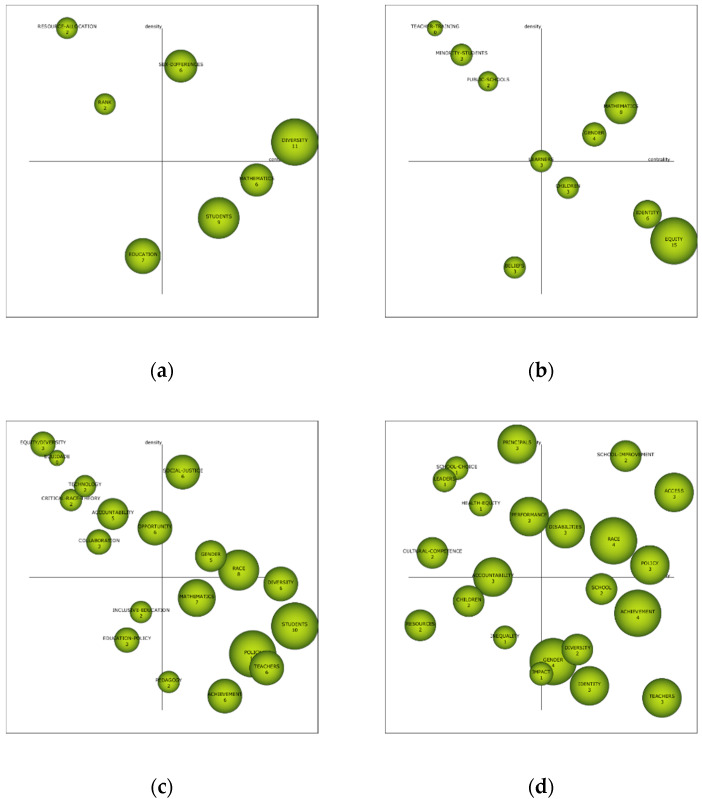
Strategic diagram of the term equity in education, based on the h-index. Note: the diagram is structured in various periods: (**a**) range 1948–2006; (**b**) range 2007–2011; (**c**) range 2012–2016; (**d**) range 2017–2019.

In the fourth period (2017–2019), the driving themes are “school-improvement”, dealing with “group-randomization-study”, “stability”, “school-evaluation”, “equity-education”, “educational-effectiveness-research”, “outcomes”, “multi-level-modelling-techniques”, and “promoting-quality”; “access”, which refers to “widening-participation”, “recognition”, “digital-divide”, “educational-opportunity”, “work”, “evaluation”, “higher-education”, and “equity”; “disabilities”, relating to “disability-studies”, “culturally-sustaining-pedagogy”, “accessibility”, “African-American”, “minority-students”, “special-education”, “inclusive-education”, and “disproportionality”; “race”, relating to “faculty”, “color”, “racism”, “department”, “experiences”, “narratives” “science”, and “women”; and “policy”; which refers to “teacher-behavior”, “of-the-art”, “comprehensive-model”, “implementation”, “student-achievement”, “organizational-routines”, “school-leaders”, and “quality-and-equity-in-education”. In addition, the themes “resources”, “children”, and “inequality” should be taken into account, because their location in the diagram places them as unknowns, that is, they may be relevant in coming years or may tend to disappear. In this period, research focuses on school improvement, access to education, student disabilities, the race of people and teacher attitudes (Figure 5).

### 3.3. Thematic Evolution of Terms

The thematic evolution shows the strength of the association established between the different themes. In this case, the relationship is established between the marked periods. The index used to establish the thematic relationship is the Jaccard index. The evolution is generated if a theme of a certain interval shares key words with the previous or subsequent interval. The more keywords have a relationship with both themes of consecutive intervals, the more solid will be their evolution. The two types of connections that can be produced are: continuous line, where its connection is thematic; and discontinuous line, whose connection is by keywords. The thickness of the lines shows the strength of the relationship between the themes.

**Figure 6:**
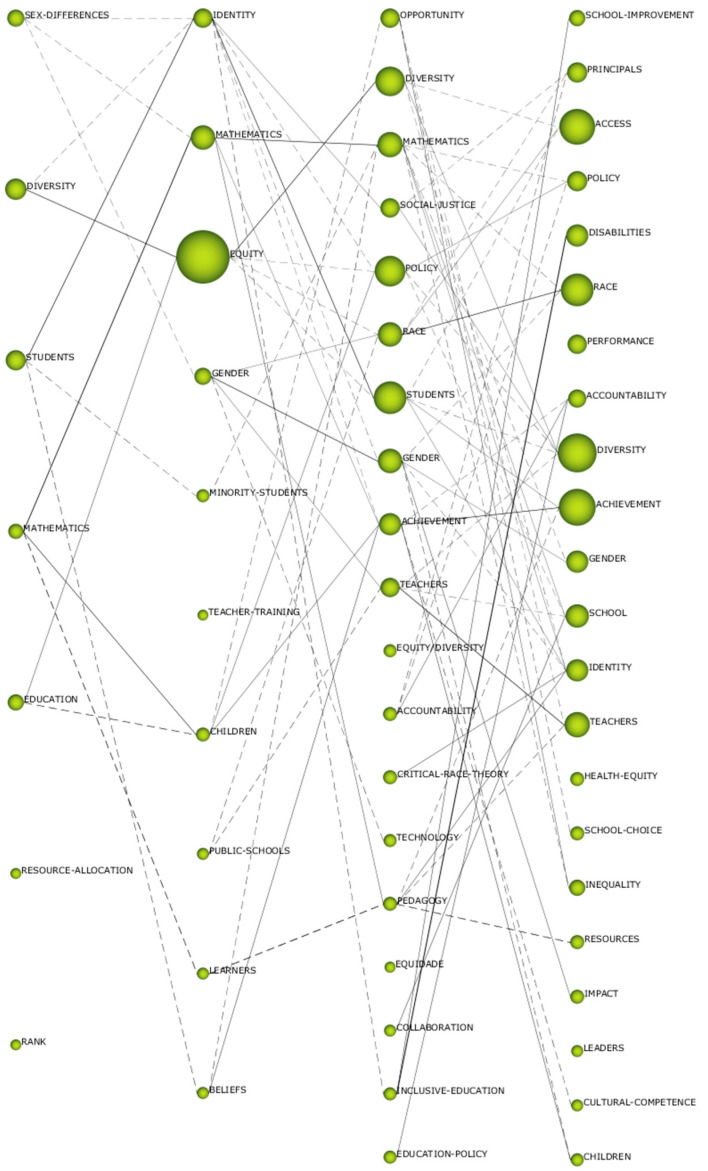
Thematic evolution by h-index.

As shown in Figure 6, the number of connections is much higher in more recent periods than in the earlier periods. Bearing in mind the dataset, there are more thematic than conceptual connections, which shows that the lines of research are related to each other. There is also a conceptual gap in the field of study, given that there is no single theme that is repeated in the four established periods. However, there are other themes, such as “mathematics”, “gender”, or “race”, that are repeated in more than one period. It should also be noted that no line of research has been established over time, nor are there any strong connections between the themes. This suggests the existing variety of research on the term equity. Finally, we observe how research trends focused in the earlier periods of study on the student’s diversity in order to evolve to more concrete and specific aspects of equity itself, such as gender and race. 

### 3.4. Authors with the Highest Relevance Index

**Figure 7:**
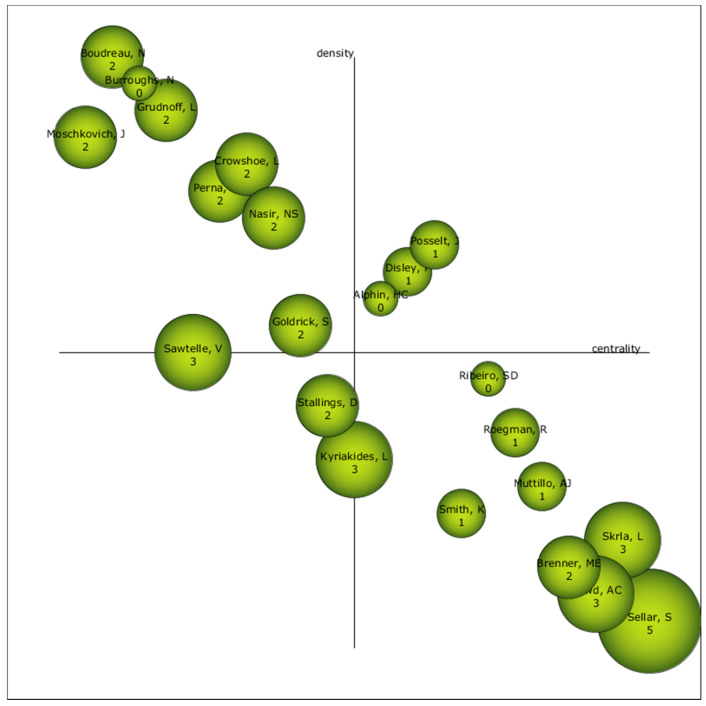
Strategic author diagram of the entire production.

Based on what is presented in Figure 7, it can be established that the most relevant authors are Posselt, J., Disley, P., and Alphin, H.C. Furthermore, Stallings, D., should be borne in mind, since his location in the diagram places him as an unknown author, given that in the near future he may be relevant or may disappear from the field of study.

## 4. Discussion

Today’s society, marked by the heterogeneity of its citizens, requires concrete actions to achieve a truly inclusive education that offers equal opportunities for all students regardless of their ethnic background, socio-economic class, or gender [2,3]. It is relevant to note that inclusive education has become an indispensable principle for dealing with students’ diversity. Moreover, it must be equitable, i.e., a system where common goods are redistributed and additional resources are provided. In this case it makes it possible for all learners have a chance of success [1]. Although equity has become an essential axis in the education system [4,5,6,7], we must consider the micro-political level that underlies the educational policies implemented: the neoliberal approach [8] or the approach that considers equity as part of social justice [9].

In any case, equity in the field of education should contribute to equal opportunities in access to studies regardless of gender [10,11], social origin, or ethnicity [12,13,14]. Equity must promote academic results and quality [15], so that students can access higher education and thus break the inequality gap [16]. Therefore, it is essential to promote compensatory educational policies (schooling, resources, or scholarships and study aids) [4] and to develop organizational and cultural aspects in educational centers [17,18,19,20,21] to encourage leadership practices [6,13,17]. The interest in creating a more equitable educational system is evident [2,26]. 

The scientific production of the term equity in education appears in an irregular way albeit constantly in the periods between 1948 and 2004. From 2005 onwards, scientific production has increased considerably. This increase was not constant, but rather irregular, given that it suffers from ups and downs in production levels. In addition, scientific production on the term equity occurs mainly in English, in the area of publication called Education Educational Research, written mostly in articles. The most productive institution in equity research is the University of California System. The author with the most production is Kyriakides and the most relevant author is Posselt. The reference journal is the Teachers College Record of the United States and the most cited document is Warschauer and Matuchniak (2010), with a total of 231 citations.

Note that the evolution of keywords is not constant at the level of production, with the percentage of keyword matching between periods falling from the first period to the last. This is interpreted as an emergence of new trends in the field of equity, of new denominational patterns created by the scientific community.

In relation to the academic performance of the different established time periods, it is observed that they do not show a consolidated theme in studies of educational equity. In fact, in the first period, the subject matter with the highest bibliometric index is found in the word “diversity”, in the second in “equity”, in the third in “policy” and “students”, and in the fourth in “race”, “achievement”, and “gender”. In other words, diachronic attention is paid to general diversity, while race and gender are analyzed as more specific elements. This shows that there is no consolidated theme in the four periods analyzed. 

Thus, there is a shift from the driving themes of “sex-differences” and “diversity” in the first period, to “mathematics” and “gender” in the second period; “social-justice”, “gender” and “race” in the third period; and “school-improvement”, “access”, “race”, “disabilities”, and “policy” in the fourth period. It is important to note that only the themes “gender” in the second and third periods and “race” in the third and fourth periods are repeated as key issues. Thus, there is a high incidence by the scientific community of analyzing differences at the personal level of individuals, especially students, focusing on gender and race.

More specifically, it is necessary to consider the topics “resources”, “children”, and “inequality”, because their location in the diagram places them as topics that in the near future may be relevant in the scientific field related to the term equity. Even more, the number of connections is very high in the more recent periods compared to the earlier ones. It is important to note there are more thematic than conceptual connections, which shows the lines of research are related to each other. However, there is a conceptual gap in the field of study, given that there is not one theme that is repeated in the four established periods. It is notable that themes such as “mathematics”, “gender”, or “race” are repeated in more than one period. On the other hand, there is no line of research based on time or with strong connections between the themes. This shows the existing variety of research on the term equity. Thus, the trend in research about the term equity is focused in the earlier periods of study on students’ diversity in order to evolve to more concrete and specific aspects of equity itself, such as, for example, gender and race.

## 5. Conclusions

In summary, the theoretical reference framework shows how equity should be incorporated into the education system under the parameters of equal opportunities, equal access to higher education, regardless of gender or socio-cultural background. In this sense, acting under the principles of equity involves developing education policies that favor compensatory education, inclusive education, and equitable education. At the macro-political level of the school organization, this means that stakeholders must create legislation, regulations, and actions that make it possible for schools to develop these equity policies. This requires human, material, and technical resources, as well as the sharing of the ideology that these principles imply and which have been set out throughout the article. Only with a joint action of the different persons responsible for the education system can educational equity be achieved, favoring the integral development of students and their incorporation into society in their lifelong learning process. 

Equity remains an outstanding issue in education for many countries in the world. It is true that recent results show a slight, but growing, improvement in equity. It is therefore necessary to identify the elements that favor and harm equity, and then to offer appropriate policies. The intention is to eliminate gender disparity in education by 2030 and ensure access to a level playing field for vulnerable people. Among these aspects we can highlight that there are countries such as Canada, Denmark, Estonia, Hong Kong, and Macao that have a balance between performance levels and equity. The United States is one of the countries where equity has improved most; immigrant students are more than twice as likely to not reach adequate levels, of which a quarter may be resistant, and attending schools where there is a majority of immigrants is not associated with worse results. A remarkable fact of equity in Spain is that there is not a high proportion of excellent students, but neither is there a high proportion of very low performance students; there may be differences in performance according to gender, due to the influence of parents, teachers, politicians, and opinion leaders, and, even, due to the differences they have in what they are good at or not, not because of what they are really capable of doing. There are examples in which equity and academic performance are not exclusive, such as high performance in countries where the relationship is less direct. Students in private schools achieve better grades than those in public schools, although if one compares their results with those of public schools according to their contexts, it is the latter that achieve better results. The greater the autonomy of schools, the greater the school performance. In high-income countries, girls finish almost all of their compulsory schooling, while in low-income countries, three out of every four girls do so. In some countries, the gender gap is less severe than the wealth and location gap; disability is another measure that leads to inequality, creating disparities; and use of the mother tongue in the early years of schooling improves student performance. Taking into account all of these aspects, education policies in different countries can improve equity aspects.

The contribution of this research consists of providing researchers with new trends on the most relevant topics for the scientific community, as well as showing a state of the art that includes the key aspects on which other research has been based. This will allow them to start from a consolidated base to initiate, develop, or guide their studies.

Finally, we note that there are several limitations in this research. Firstly, there is the debugging of the data presented in WoS, where repeated documents are presented or which are not related to the subject matter of the study. Secondly, the establishment of the intervals, in this case, is a question of equity, given that we sought at all times to maintain a similar number of papers in each of the intervals. Thirdly, and finally, the parameters marked in this study were established according to our criteria. Thus, results are presented in accordance with their size and relevance. In this way, the data shown here should be analyzed with caution, because the change in the parameters established in this research may influence the number of topics presented and their connections. As future lines of research, it is proposed to develop practical applications and pedagogical actions in the educational field focused on education and ethics.

## Figures and Tables

**Table 1:** Production indicators and inclusion criteria.

Configuration	Values
Analysis unit	Keywords authors, keywords WoS
Frequency threshold	Keywords: P_1_ = (2), P_2_ = (2), P_3_ = (2), P_4_ = (2)
	Authors: P_X_ = (2)
Network type	Co-occurrence
Co-occurrence union value threshold	Keywords: P_1_ = (2), P_2_ = (2), P_3_ = (2), P_4_ = (2), P_5_ = (2)
	Authors: P_X_ = (2)
Normalization measure	Equivalence index
Clustering algorithm	Maximum size: 9; Minimum size: 3
Evolutionary measure	Jaccard index
Overlapping measure	Inclusion Rate

**Table 2:** Scientific language used.

Language	EQUI
English	2488
Spanish	90
Portuguese	15

**Table 3:** Areas of knowledge.

Publication Area	EQUI
Education Educational Research	2408
Education Scientific Disciplines	161
Psychology Educational	88
Education Special	73
Social Issues	46

**Table 4:** Type of document.

Document Type	EQUI
Article	1741
Book Chapter	473
Book Review	264
Editorial Material	256
Proceedings Paper	244

**Table 5:** Institutions.

Organization	EQUI
University of California System	125
California State University System	53
University of North Carolina	49
University of Texas System	49

**Table 6:** Most prolific authors.

Authors	EQUI
Kyriakides, L.	16
Charalambous, E.	13
Ainscow, M	10
Creemers, B	9

**Table 7:** Source of origin.

Source	EQUI
Teachers College Record	46
Phi Delta Kappa	45
Educational Policy	37
Educational Leadership	33

**Table 8:** Country.

Countries	EQUI
United States	1371
England	201
Australia	191
Canada	135

**Table 9:** EQUI: most cited articles.

References	Institution First Author	Country First Author	Research Method Used	Quotes
[37]	University of California Irvine	USA	Qualitative	231
[38]	University of Massachusetts System	USA	Qualitative	199
[39]	Indiana University System	USA	Qualitative	183
[40]	McGill University	Canada	Qualitative	153

**Table 10:** Thematic performance in EQUI.

**Interval 1948–2006**						
**Denomination**	**Works**	**Index h**	**Index g**	**Index hg**	**Index q^2^**	**Citations**
Sex-differences	8	6	7	6.48	19.6	363
Diversity	12	11	12	11.49	22.25	520
Students	11	9	10	9.49	19.44	564
Mathematics	6	6	6	6	17.32	346
Education	7	7	4	7	14.73	297
Resource-allocation	2	2	2	2	7.21	31
Rank	2	2	2	2	3.13	9
**Interval 2007–2011**						
**Denomination**	**Works**	**Index h**	**Index g**	**Index hg**	**Index q^2^**	**Citations**
Identity	10	6	7	6.48	18.97	309
Mathematics	15	8	10	8.94	21.54	394
Equity	43	15	26	19.75	21.94	723
Gender	8	4	6	4.9	7.48	61
Minority-Students	4	3	3	3	9.8	245
Children	5	3	4	3.46	9.17	82
Public-School	3	2	3	2.45	10.77	86
Learners	3	3	3	3	8.31	136
Beliefs	3	3	3	3	7.14	48
**Interval 2012–2016**						
**Denomination**	**Works**	**Index h**	**Index g**	**Index hg**	**Index q^2^**	**Citations**
Opportunity	10	6	9	7.35	9.17	98
Diversity	20	6	16	9.8	15.68	256
Mathematics	16	7	12	9.17	9.9	147
Social-justice	10	6	8	6.93	9.17	83
Policy	21	10	17	13.04	14.49	295
Race	15	8	11	9.38	12	158
Students	23	10	18	13.42	15.81	361
Gender	15	5	10	7.07	9.75	103
Achievement	13	6	10	7.75	9.8	112
Teachers	10	6	9	7.35	6.93	84
Equity/diversity	4	3	3	3	3.46	14
Accountability	5	5	5	5	7.75	84
Critical-race-theory	5	2	3	2.45	10	72
Technology	4	2	2	2	9.9	62
Pedagogy	5	2	4	2.83	5.29	27
Collaboration	3	3	3	3	6.71	42
Inclusive-education	4	2	4	2.83	4.69	19
Education-policy	4	3	3	3	4.58	21
**Interval 2017–2019**						
**Denomination**	**Works**	**Index h**	**Index g**	**Index hg**	**Index q^2^**	**Citations**
School-improvement	7	2	3	2.45	4	14
Principals	11	3	6	4.24	5.2	37
Access	26	3	6	4.24	6.24	45
Policy	11	3	3	3	3	15
Disabilities	13	3	4	3.46	4.24	25
Race	23	4	6	4.9	5.66	54
Performance	10	3	6	4.24	4.74	45
Accountability	9	3	4	3.46	3.46	21
Diversity	29	2	3	2.45	3.46	21
Achievement	27	4	4	4	4.47	36
Gender	13	4	5	4.47	5.29	39
School	14	2	3	2.45	3.46	16
Identity	13	3	4	3.46	4.9	25
Teachers	16	3	5	3.87	5.2	37
Health-equity	5	1	1	1	1.41	3
School-choice	5	1	2	1.41	1.73	4
Inequality	7	1	2	1.41	2.24	7
Resources	5	2	3	2.45	3.16	10
Impact	5	1	2	1.41	2.83	9
Leaders	3	1	1	1	1.41	3
Cultural-competence	3	2	3	2.45	4	13
Children	4	2	4	2.83	4.9	22
